# Molecular characteristics and replication mechanism of dengue, zika and chikungunya arboviruses, and their treatments with natural extracts from plants: An updated review

**DOI:** 10.17179/excli2019-1825

**Published:** 2019-10-31

**Authors:** Anny Karely Rodriguez, Ana Luisa Muñoz, Nidya Alexandra Segura, Héctor Rafael Rangel, Felio Bello

**Affiliations:** 1 Faculty of Science, Universidad Antonio Nariño (UAN), Bogotá, 110231, Colombia; 2 Faculty of Science, Universidad Pedagógica y Tecnológica de Colombia, Tunja 150003, Colombia; 3 Laboratory of Molecular Virology, Instituto Venezolano de Investigaciones Científicas, Caracas, 1204, Venezuela; 4 Faculty of Agricultural and Livestock Sciences, Program of Veterinary Medicine, Universidad de La Salle, Bogotá, 110131, Colombia

**Keywords:** Dengue virus, Zika virus, Chikungunya virus, viral replication, viruses biology, natural extracts

## Abstract

Viruses transmitted by arthropods (arboviruses) are the etiological agents of several human diseases with worldwide distribution; including dengue (DENV), zika (ZIKV), yellow fever (YFV), and chikungunya (CHIKV) viruses. These viruses are especially important in tropical and subtropical regions; where, ZIKV and CHIKV are involved in epidemics worldwide, while the DENV remains as the biggest problem in public health. Factors, such as, environmental conditions promote the distribution of vectors, deficiencies in health services, and lack of effective vaccines, guarantee the presence of these vector-borne diseases. Treatment against these viral diseases is only palliative since available therapies formulated lack to demonstrate specific antiviral activity and vaccine candidates fail to demonstrate enough effectiveness. The use of natural products, as therapeutic tools, is an ancestral practice in different cultures. According to WHO 80 % of the population of some countries from Africa and Asia depend on the use of traditional medicines to deal with some diseases. Molecular characteristics of these viruses are important in determining its cellular pathogenesis, emergence, and dispersion mechanisms, as well as for the development of new antivirals and vaccines to control strategies. In this review, we summarize the current knowledge of the molecular structure and replication mechanisms of selected arboviruses, as well as their mechanism of entry into host cells, and a brief overview about the potential targets accessed to inhibit these viruses *in vitro* and a summary about their treatment with natural extracts from plants.

## Introduction

Arbovirus (arthropod-borne viruses) is a group that comprises viruses which belong to four different families: Togaviridae, Reoviridae, Flaviviridae, and Bunyaviridae, these are characterized by a cycle of transmission between vertebrate hosts and arthropod vectors and are responsible for the development of emerging and re-emerging diseases worldwide in recent decades (Hubálek et al., 2014[30]; Weaver and Reisen, 2010[95]). The Aedes (Stegomyia) aegypti (L.) and Aedes (Stegomyia) albopictus (Skuse) mosquitoes (Diptera: Culicidae) are primary vectors for arboviruses such as dengue, yellow fever, zika and chikungunya viruses guaranteeing their permanence and circulation in the population (Leta et al., 2018[38]). Some of these viruses are involved in epidemics in the African continent and Latin America, with possible urban cycles (Bhatt et al., 2013[10]; Colón-González et al., 2017[20]; Higuera and Ramírez, 2019[29]; Litvoc et al., 2018[44]). 

According to the Pan American Health Organization reports, the vector-borne diseases are ordered by frequency and prevalence in the following manner: 1^st^ place dengue, with cases in almost all of the countries of the American region; 2^nd^ chikungunya; 3^rd^ zika, which is then followed by Malaria, Chagas, Leishmaniasis and Yellow fever (PAHO-CHA-IR, 2016[57]). 

It is important, from the public health point of view, to deepen the studies related to dengue virus (DENV), yellow fever virus (YFV), zika virus (ZIKV) and chikungunya virus (CHIKV) that lead to the development of effective, specific and economic therapeutic strategies for their control. 

In Latin America, one of the most important arbovirus families is *Flaviviridae*, which comprises more than 70 species. This family is constituted by four genera: *Hepacivirus*, one of the representatives being the hepatitis C virus (HCV); *Pestivirus*, for which Bovine viral diarrhea virus (BVDV) is a representative; *Pegivirus,* to which belongs the viruses GBV-A, GBV-D, GBV-C, among others; and the genus *Flavivirus*, which is one of the largest genus, and several medically important species such as YFV, DENV, and ZIKV, among other arboviruses, belong to this genus (Blitvich and Firth, 2017[11]). 

One of the first species described in the genus *Flavivirus* was the YFV; it was isolated for the first time in 1927 in Ghana. The estimated number of cases of YFV in Africa and Latin America are about 120,000 per year, with approximately 45,000 deaths/year. The clinical manifestations range from an asymptomatic disease (around 50 % of the cases) to a very severe form of the disease, with 20 % mortality rate (Litvoc et al., 2018[44]). Currently, its prevention has been achieved via the 17D vaccine, however it still remains a public health problem, due to the current outbreaks in endemic and non-endemic areas (Douam and Ploss, 2018[21]), which raised interest in relation to the potential impact of YFV into non-endemic countries, where usually people do not get vaccinated.

Another related *Flavivirus* is dengue virus, one of the main public health problems in tropical and subtropical regions. There is an alarming estimation of 390 million of dengue infections per year, with distribution in almost all countries of the American region. The clinical manifestations vary from asymptomatic or mild fever to severe dengue (hemorrhagic and shock syndromes), with a mortality rate of up to 20 % among the patients with severe dengue (Bhatt et al., 2013[10]; PAHO-CHA-IR, 2016[57]). Dengue fever may be caused by four different serotypes: DENV1, DENV2, DENV3, or DENV4 (Kostyuchenko et al., 2013[37]), where the immunity towards the infecting serotype is life-long; however, severe forms of the disease may occur after the second or subsequent infection caused by a different dengue virus serotype, since the cross-protection against other serotypes is limited (Martina et al., 2009[47]; Torres et al., 2017[87]). Recently a fifth serotype has been published, however it seems to be limited to the sylvatic cycle only (Mustafa et al., 2015[52]). The development of vaccines against dengue has not been easy, perhaps due to the genomic variability of its four serotypes and the complexity of its pathogenesis. Currently there is an approved vaccine: CYD-TDV (under the commercial name Dengvaxia®), developed by Sanofi Pasteur, however there is still no clarity about its safety and efficacy (Malisheni et al., 2017[46]; Prompetchara et al., 2019[62]). 

ZIKV is a public health problem since it caused a pandemic between 2015 and 2016, with more than one million cases reported only in Latin America (Hennessey et al., 2016[28]). Two genotypes have been described (African and Asian), based on the phylogenetic analysis (Shi and Gao, 2017[72]). Its infection may be asymptomatic or may present acute fever and rash; the reports have associated the infection with congenital syndromes and severe neurological complications (Musso and Gubler, 2016[51]). These neurological complications can be a consequence of the activation of protein complexes involved in the proliferation and apoptosis processes of glial cells. Although ZIKV presents a mature structure similar to other known flaviviral structures (Shi and Gao, 2017[72]; Wang et al., 2017[92]), some of its clinical manifestation differs from other flaviviruses. Additionally, the genomic divergences among the ZIKV strains/isolates can in turn be associated with the differential clinical manifestations of the infection (Shi and Gao, 2017[72]; Wang et al., 2017[92]). 

Another important arbovirus family found in Latin America is *Togaviridae. *The CHIKV belongs to this family, and the *Alphavirus* genus. Currently there are four genotypes of chikungunya reported: The East-Central-South Africa, West Africa, Asian, and Indian Ocean lineage; all of them were named based in their geographic distribution. The CHIKV has a close relation with more alphaviruses such as: The o´nyong'nyong Virus, Rio Ross Virus, Mayaro Virus, Barmah Forest Virus and the group of Sindbis Virus, all of them are known as causative agents of arthritis (Vu et al., 2017[91]). Since 1950, CHIKV was found and characterized only in East Africa; only in the late 1990s and early 2000s, CHIKV remerged on a global scale (Ganesan et al., 2017[25]), arriving to the Americas in 2013 affecting more than one million people (Vu et al., 2017[91]). Regarding the clinical manifestations, most patients suffer a self-limited febrile syndrome with polyarthritis and rash; however, some patients have reported persistent arthralgia even for years. Alterations of the central nervous system such as convulsions, meningoencephalitis, and Guillain-Barré syndrome have been reported. Myocarditis, liver failure in patients with antecedents of chronic liver disease, endocrine alterations, pancreatitis, and respiratory failure have also been reported, although less frequently (Simon et al., 2011[77]). 

The prevalence of these viruses is guaranteed by the proximity of populations to the jungle areas that allows the maintenance of all these viruses that move back and forth between each environment (Figueiredo, 2019[24]), this complex situation is probably the cause of some of the reemerging epidemics, related to some arboviruses.

Molecular analysis is important to understand the mechanisms by which arboviruses cause infectious and remain in nature, as well as its pathogenicity and immunogenicity, opening a path for new research. This review is focused on the genomic structure, replication mechanisms of some *Flavivirus* and *Alphavirus* and a summary about their possible treatments with natural extracts from plants.

## Molecular Structure of Flavivirus and Alphavirus

### Flaviviruses

All viruses included in the *Flavivirus *genus have some common characteristics: a size of 40-60 nanometers; an envelope that is covering an icosahedral nucleocapsid, which protects the genetic material; and a single positive sense RNA strand, which is approximately 10.000 to 11.000 bases in length. This strand has a unique open reading frame (ORD) consisting of approximately ~3400 codons, that encodes for a unique viral polyprotein (Brinton and Basu, 2015[15]). These viruses show a type I cap in the 5'-end. The presence of a methylation in 5' cap in the RNA viral genome has an important role to play in the viral translation/replication process and is involved in the evasion of the immune reactions that restrict the viral replication in the host. Presence of Poly (A) tails at 3'-termini, in the 5' and 3' terminal regions, there are short conserved sequences whose functions are still unknown. The *Flavivirus *genome works as a viral mRNA and as a template for the synthesis of small RNA chains. Its organization is maintained between the different genera (Lindenbach and Rice, 2003[43]; Brinton and Basu, 2015[15]). 

**Figure 1:**
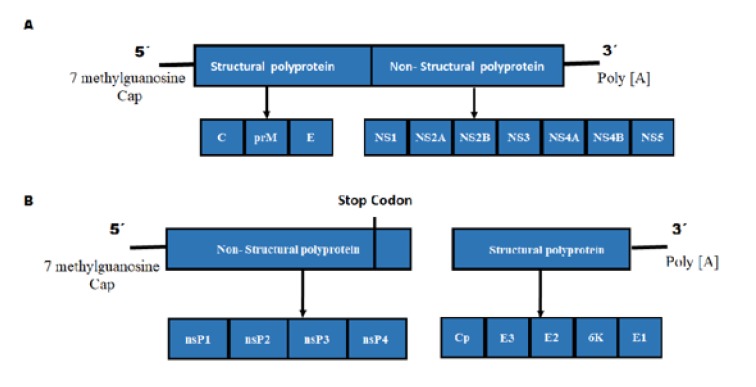
Comparison of Flavivirus and Alphavirus genome. (A) The Flavivirus, similar to the Alphavirus genus, contain single stranded RNA of positive sense; however, this has a unique open reading frame (ORF). In the 5´ end, a type I cap is present and at the 3´ end, a polyadenylation tail is observed. A typical genome has a similar order of genes and some conserved non-structural proteins (NS). (B) The viruses belonging to the Alphavirus genus possess a genome consisting of a positive sense single stranded RNA with two ORFs at the 5´ end. These code for four non-structural protein (nsP1-4). Another ORF at the 3´ end, codes for five structural proteins namely, capsid protein (C), envelope glycoproteins (E1 and E2), and two small cleavage products (E3 and 6K).

In the family *Flaviviridae*, most of the members have the typical genome with a similar order of genes and some conserved non-structural proteins (Figure 1(Fig. 1)). However, they diverge notoriously in the *cis*-acting regulatory elements of the RNA located at their 3' and 5' ends. For example, the polyproteins translated from the genomes of *Hepacivirus, Pestivirus,* and *Pegivirus* begin with an AUG sequence that is found at the 3' end. This is the internal ribosome entry site (IRES). On the contrary the translated protein of *Flavivirus* is dependent on a type I cap located at the 5' end. The genome of the *Flaviviridae* family is organized as follows: 

Flavivirus: 5'CAP (I)-5'UTR-C-prm-E-NS1-NS2A-NS2B-NS3-NS4A-NS4B-NS5-3'UTR 

The ORF of the *Flavivirus* encodes for a polyprotein which is processed by host and viral proteases in three structural proteins, the C protein of nucleocapsid, a glycoprotein precursor of prM membrane, and a glycosylated envelope protein E, along with seven non-structural proteins (NS) viz. NS1, NS2A/B, NS3, NS4A, NS4B, and NS5, which are arranged from terminal N to C. The process of proteolysis of the polyprotein of *Flavivirus* is carried out through the combination of four different proteases (Bollati et al., 2010[12]).

### Processing of the viral polyprotein

1. A host signal peptidase process the anchor junctions -C/prM, prM/E, E/NS1, and NS4A-“2K”/NS4B.
2. The viral NS3 serine protease needs NS2B as a cofactor, to process the C-anchor junctions of NS2A/NS2B, NS2B/NS3, NS3/NS4A, NS4A/"2K", and NS4B/NS5.
3. The furin of the host, or other furin binding proteins process the binding between pr/M.
4. The proteases of the host involved in separating the NS1/NS2A junction are unknown.

Among the structural proteins, the C protein of nucleocapsid protects and encapsulates the genetic material. An envelope glycoproteins prM/M creates the superficial structure of the virion. The protein prM/M has an important role in the maintenance of the spatial structure of the E protein, which is composed of three different structures, envelope domain I, II, and III (EDI, EDII, EDIII, respectively), connected to the viral membrane (Zhang et al., 2017[102]). In YFV, the E protein is highly immunogenic and plays a central role in viral binding and fusion (Volk et al., 2009[90]). 

EDI forms the central domain of the envelope protein, which establishes the protein direction and its glycosylation sites. The glycosylation domains of EDI are associated with the viral production, pH sensitivity and neuroinvasiveness in most flaviviruses. Interestingly, ZIKV has a single glycosylation site (N154) in the EDI with a longer loop (residues 145-160) compared to other flaviviruses (Kostyuchenko et al., 2013[37]). EDII plays an important role in the membrane fusion, and EDIII is the main target of the neutralizing antibodies; these proteins are essential in the biology of the *Flavivirus* (Shi and Gao, 2017[72]; Zhang et al., 2017[102]). The structure of the Domain III of YFV differs from the structure of other mosquito-transmitted flaviviruses, specifically in the surface exposed BC loop, since it is one amino acid shorter, than even other mosquito-borne and non-vector borne viruses. This change can modify neutralization sites and receptor interactions, and therefore, the viral pathogenesis (Volk et al., 2009[90]).

A difference in the amino acid sequence of the EDIII region has been reported in the DEN4 sylvatic and human strains, which may be related to their jump from a sylvatic host to a human one (Volk et al., 2007[89]). Therefore, this region among *Flavivirus* shows considerable variation.

The non-structural proteins, modulate intracellular process, such as viral replication, assembly, proteolysis, maturation, and regulation of the host immune response and are involved in the evasion of the immune response. In the case of evasion of the immune response by DENV the following are involved: NS2A, NS2B3, NS4A, NS4B and NS5 (Chen et al., 2017[19]). 

About the non-structural proteins, they change in functions and structure between different Flaviviruses. For example, NS1 is a highly conserved protein that is encoded exclusively by the members of genus *Flavivirus* and is considered as the major antigenic marker during infection. This non-structural protein is a dimeric protein with molecular weight between 46-55 kDa depending on the degree of glycosylation, which is important for the efficiency of the viral secretion, replication, and virulence. Thus, deletion of NS1 glycosylation sites of DENV and YFV, significantly reduce their viral replication. NS1 can exist as a monomer, dimer (protein connected to the mNS1 membrane) and also as a hexamer (secreted protein, sNS1) (Rastogi et al., 2016[66]; Chen et al., 2017[19]). A recent study shows that the overall structure of NS1 is similar among YFV, ZIKV, DENV, and WNV (Wang et al., 2017[94]). NS1 of ZIKV has a diversity of electrostatic surface features in the host-virus interaction context as well as unique dimeric assembly (Song et al., 2016[80]). 

The NS1 function is to activate the Toll-like receptors (TLRs) and inhibit the complement system in order to interact with the NS4 protein. The activation of the TLRs is important as it leads to the activation of transcription factors and the production of cytokines as a part of the innate immune response. The main receptor for *Flaviviridae* is TLR3, which in turn can modulate the activation of dendritic cells, macrophages, and B cells as well as triggers the IFN mRNA transcription, thus inducing the innate antiviral responses (Alayli and Scholle, 2016[3]; Chen et al., 2017[19]). Spontaneous mutation resulting in a single amino acid substitution in ZIKV NS1 protein increased its infectivity in *Aedes aegypti* mosquitoes, which most likely had promoted its transmission in the recent epidemic (Liu et al., 2017[45]). 

In the case of WNV, NS1 reduces the regulation of the IFN-β and IL-6 transcription mediated by TLR3; nonetheless, this inhibition can be dependent on cell type (Chen et al., 2017[19]). It has been reported that the expression of the WNV, YFV or DENV-2 NS1 protein, does not alter the TLR3 translation levels in two cell lines (HeLa and HEK-293) (Baronti et al., 2010[6]). This could indicate that the TLR3 pathway is not exclusive in the control of flaviviruses replication and they possess other mechanisms for evading the host immune response. 

On the other hand, in the dengue virus, NS1 anchors the replication complex to the host endoplasmic reticulum membrane, and interacts directly with NS4B, As a result, it plays an important role in the viral replication process. Furthermore, YFV and DENV are associated with hemorrhagic fever, this may be due to the increased vascular permeability. However it can be mediated by the NS1 protein, because it has been shown that its binding to heparin sulfate of the endothelial glycocalyx layer may alter the capillary permeability (Puerta-Guardo et al., 2016[64]). This is also relevant in the severe forms of dengue since anti-NS1 antibodies join to the surface of the endothelial cells and platelets producing cell damage (Chen et al., 2018[18]). Besides, ZIKV NS1, from Asian lineage strains, can be involved in the developmental complications such as microcephaly in fetuses and also severe neurological manifestations in adults. However, currently it is not clear how the virus can penetrate the hematoencephalic barrier (Wang et al., 2017[93]; Xia et al., 2018[98]). However, an alternative protein, the NS1', has been reported in ZIKV (specifically Asian lineage) and other neuroinvasive* Flavivirus. *This suggests that this protein may be involved with the viral neurotropism and therefore with its neuroinvasiveness (Tambonis et al., 2017[82]). 

As a consequence about the previously reviewed information, it is possible to establish that the NS1 protein of the *Flavivirus*, induces a protective immune response against infection; it is an important immunogen in the viral infection course (Chen et al., 2017[19]), and a biomarker for the diagnostics of dengue, zika and chikungunya infections (Cecchetto et al., 2017[17]).

The NS2 protein, is encoded by the NS2 region of the *Flaviviridae* genome. The NS2 protein can be also processed into the matured proteins such as NS2A and NS2B (Chen et al., 2017[19]). The NS2A is a 226 amino acid long hydrophobic protein, that is inserted in the membrane and interacts specifically with negatively charged phospholipids. This protein contains some transmembrane domains and is associated with the membrane of host endoplasmic reticulum (RE). It is involved in the evasion of the immune response since it suppresses Interferon β transcription (IFN-β), and as a result, the interferon response, which is one of the host's first lines of defense. The NS2A is required for the processing of NS1 containing specific recognition motifs targeted by specific proteases. It is also involved in the virion assembly and the synthesis of viral RNA. For example, in the case of YFV, a Lys-to-Ser mutation at position 190 in NS2A blocked the production of virus particles (Leung et al., 2008[39]). Additionally, it has been reported that in DENV there are two different sets of NS2A molecules, which could be interesting for the development of control strategies (Xie et al., 2015[99]). The functions of NS2A are related to the membrane structure and orientation, and mainly in both viral RNA synthesis and virion assembly (Murray et al., 2008[50]; Xie et al., 2015[99]). NS2B is an integral protein of 14 kDa and is composed of three domains, two transmembrane segments located at the N and C terminal regions, and a central region of 47 amino acids that acts as cofactor for the protease NS3. The complex NS2B-NS3 serves as a place for the assembly of the replication complex of the flaviviruses and modulates both the viral pathogenesis and the immune response of the host (Murray et al., 2008[50]; Chen et al., 2017[19]). Although the functions of NS2A and NS2B proteins are not well understood, it is known that in DENV, apoptosis is induced by NS2B-NS3 protease precursor and NS3 protease, most likely through the caspase-8 pathway or NF-κB pathway (Uno and Ross, 2018[88]). 

NS3 is an enzyme that shows approximately 65 % sequence identity between DENV, ZIKV, and YFV (Brand et al., 2017[13]), therefore is interesting for the development of control strategies. The NS3 protein is the second largest protein after NS5, and is composed by two regions: the N-terminal region that contains a serine protease domain and a chymotrypsin protease domain. The C-terminal region that contains both a nucleoside triphosphatase (NTPase) and a RNA helicase domain. Serine protease is involved in the proteolytic processing of the viral polyprotein in three single proteins, as mentioned above, and thus plays a central role in the viral replication cycle. The chymotrypsin protease cleaves the viral precursor polyprotein to release each individual NS protein and the NTPase/dependent RNA helicase, which are involved in the replication of the genome and synthesis of viral RNA (Natarajan, 2010[54]). Likewise, DENV-NS2B is required as a cofactor for NS3 protease activity, for example, it has been described that in DENV is responsible for the cleavage between NS2A/2B, NS2B3, NS3/4A and NS4B/5 (Chen et al., 2017[19]).

DENV NS4 protein is cleaved by the NS3 host signal protease and peptidase, producing mature NS4A and NS4B proteins. This process is necessary for the inhibition of the IFN signaling pathway, as well as for the viral replication process (Zou et al., 2015[104]; Chen et al., 2017[19]). NS4A is a small integral membrane protein and it contains four transmembrane segments that act as a scaffold for the replication complex. It has been proposed that NS4A leads to membrane modifications and plays an important role in the formation of structures derived from the host's membrane. The N-terminal region of NS4A interacts specifically with curved membrane regions. It is an essential component of the viral replication cycle because it interacts directly with the vimentin protein of the cytoskeleton, an event that is necessary for the correct localization of the replication complex in the perinuclear region (Teo and Chu, 2014[84]). On the other hand, the protein NS4A is involved in the induction of autophagy as a mechanism for infection (McLean et al., 2011[48]). NS4B is formed by three C-terminal trans-membrane segments and is involved in blocking the signaling transduction of the IFN-α/β, as well as modulating the function of NS3 helicase. In YFV and WNV, NS4B and STAT1 show cytoplasmic localization, and can inhibit the 'Interferon Stimulated Response Element' activation mediated by IFNβ (Nemésio et al., 2012[55]; Uno and Ross, 2018[88]). Even though the NS4B protein is highly conserved among the DENV serotypes, mutations in the NS4B of YFV can modulate its neuro-invasion capacity, as well as its neuro-virulence (Zmurko et al., 2015[103]). During Zika infection, NS4A and B are involved in the induction of autophagy in the fetal neural stem cells leading to defective neurogenesis (Liang et al., 2016[41]).

Finally, NS5, a 104 kDa protein is encoded by the C terminus of *Flavivirus* genome. It is highly conserved among the *Flavivirus*, with a homology of about 68 % between DENV, ZIKV and YFV (Potisopon et al., 2014[61]; Brand et al., 2017[13]). NS5 is composed of two domains: the methyltransferase domain, indispensable for the formation of the CAP structure of the viral genome, and the RNA-dependent RNA polymerase domain, which is necessary for the RNA genome replication process. The NS5 protein of the *Flavivirus* can inhibit the IFN signals during the infections caused by DENV, YFV, ZIKV, and HCV, in order to evade innate immunity; this is achieved through the interaction with STAT2 protein or by modulating RNA splicing within the host cell (Murray et al., 2008[50]). DENV infected cells show localization of NS5 protein mainly in the nucleus; this may be related to the modulation of the cytokine gene expression (El Sahili and Lescar, 2017[23]). The NS3 protein initially cleaves the NS5 in HCV, producing the NS5A and NS5B mature proteins. NS5B is an RNA-dependent RNA polymerase involved in the viral replication, with a terminal nucleotide transferase activity using uridine triphosphate as a substrate. Because the NS5 is a highly conserved protein among* Flavivirus*, and its important role during the viral replication and evasion of the host immune system, has led to the proposal of the NS5 as a target for the development of antivirals against *Flavivirus* infections (El Sahili and Lescar, 2017[23]; Shi and Gao, 2017[72]). 

### Alphaviruses

The *Alphavirus* genus, consisting of about 29 virus species, is characterized by an icosahedral symmetry and a genome that consists of a positive sense single-stranded RNA of approximately 11.8 Kb length. This ssRNA is composed of two open reading frames (ORFs), the one on the 5' end produces non-structural proteins (nsP1-4), and the second one at the 3' end that produces five structural proteins called: capsid protein (C), envelope glycoproteins (E1 and E2), and two small cleavage products (E3 and 6K). The 5' end of the CHIKV genome also shows a 7-methylguanosine cap (Figure 1(Fig. 1)) (Jose et al., 2009[31]; Shi and Gao, 2017[72]).

The structural proteins are required for entry, assembly, and budding steps. These proteins are processed as a polyprotein, which are cleaved by capsid and host proteases into C, E3, E2, 6K, and E1 proteins. The proteins E3 and E2 have pE2 as intermediary compound, cleaved by host furin protease. The N-terminal domain of E3 is an uncleaved leader peptide for E2, which may help shield fusion peptide in E1 during egress. The E1 glycoprotein mediates the virus-membrane fusion. The E2 mediates the binding to the receptors and attachment factor on the cell membrane and is the major target for neutralizing antibodies. At low pH E1 and E2 dissociated in the endosome, which trigger the fusion of the viral and endosomal membranes, thus releasing the viral genome into the cytoplasm (Li et al., 2010[40]). It has been reported that E2 residue 82, in CHIKV, is determinant of glycosaminoglycan utilization and therefore, in the viral entry, this may have had an impact on the attenuation of the vaccine strain 181/25 (Silva et al., 2014[76]).

Besides, the protein 6K acts as a leader peptide for E1 and facilitates particle morphogenesis, and are involved in the formation of viroporins that play a role in the release of virus progeny, the intracellular trafficking of glycoprotein and the permeability of the membrane. The transframe protein, generated by ribosomal frame shifting, shares N-terminus with 6K, which is also considered as a putative ion channel that may enhance particle release. E1 in CHIKV is a 436 aa type II fusion protein, through fusion of viral envelope and endosomal cellular membrane (Jose et al., 2009[31]; Bautista-Reyes et al., 2017[9])_._

In turn, capsid protein has two domains: The N-terminal RNA binding domain and the C-terminal protease domain. The N-terminal region is indispensable for binding to RNA genome, C protein multimerization and its subsequent nuclear/cytoplasmic translocation. About the nuclear translocation is known that induces host cell transcriptional blockade in encephalitic alphaviruses, however it is not clear in arthritogenic alphaviruses yet (Taylor et al., 2017[83]), however mutation of a nucleolar localization sequence in the CHIKV N-terminal region of capsid protein, is involved in viral attenuation. Thus, this motive capsid protein is a target for the development of vaccines (Taylor et al., 2017[83]; Yap et al., 2017[100]; Sharma et al., 2018[71]). 

On the other hand, the alphaviruses present four non-structural proteins (nsP), which are well characterized in CHIKV (Pietilä et al., 2017[59]; Silva and Dermody, 2017[75]):

- nsP1 have both guanine-7-methyltransferase and guanylyltransferase activities, thus is responsible for the addition of the 5´Cap to viral genomic and subgenomic RNAs, a step necessary to avoid the viral RNA degradation and for its subsequent translation. This protein is also involved in anchoring the replications complexes to the cellular membranes maybe through interaction with anionic phospholipids from the membrane (Abu Bakar and Ng, 2018[2]; Mutso et al., 2018[53]).
- The master regulator of the Alphavirus cycle, the nsP2, which consists of 798 aa. In the c-terminal region shows a papain-like cysteine protease, which is required to process P1234 polyprotein into mature non-structural proteins, act as a RNA triphosphatase involved in viral RNA capping reactions, as a nucleotide triphosphatase supply the RNA helicase activity. Mature nsP2, in its N-terminal region contains a helicase domain, catalyzes the cleavages between the nonstructural proteins, and an inactive RNA methyltransferase-like domain. The C-terminal subdomain is an 5-adenosyl-L-methionine-dependent (SAM) RNA methyltransferase domain with no enzymatic function (Rana et al., 2017[65]; Mutso et al., 2018[53]). Virus with mutations in this nsP2 protein are unable to produce cytopathic effect (Shin et al., 2012[73]).
- nsP3, which consists of 530 aa, have three domains: the N-terminal macro domain, the central zinc-binding domain and the C-terminal hypervariable domain (HVD). This protein is involved in the negative sense and subgenomic viral RNA synthesis and is a component of the viral RNA replicase complex and has been associated with the neurovirulence of some alphavirus. The N-terminal domain conserved among alphaviruses, which exhibits both ADP-ribose and RNA binding activities, and also dephosphorylate ADP-ribose-1′′-phosphate and have de-ADP-ribosylating activity. The central zinc-binding domain is also conserved among alphaviruses, it is involved in the RNA replication, however it is no yet cleared. The hypervariable domain shows variability even between closely related alphaviruses, being indispensable for alphavirus replication (Götte et al., 2018[27]). Mutations in nsP3 have been associated with defects in the initiation of minus-strand synthesis or subgenomic RNA synthesis (Mutso et al., 2018[53]), and the degradation of this protein seems to be involved in the upregulation of nsP4 levels (Götte et al., 2018[27]).
- The highly conserved protein nsP4 has a homology greater than 50 % among alphaviruses. This protein consists of 611 aa, is a RNA-dependent RNA polymerase and terminal adenosine transferase, responsible for synthesis of different viral RNAs and adding (and repair) the poly (A) tails to positive strand RNAs, essential steps to the virus replication (Tomar et al., 2006[86]; Mutso et al., 2018[53]).

## Replication of Flavivirus and Alphavirus

After an infected mosquito feed on a mammalian host, the viral particles are released in the bloodstream and then infect the white cells; the specific binding site is located in the E protein of the virus (Allison et al., 1995[5]; Welsch et al., 2009[97]).

Both flaviviruses and alphaviruses enter cells through clathrin-coated vesicles or specific receptors on the cell membrane. However, in all the cases the membrane fusion process is triggered by the low pH of the endosome. E and E1 proteins, from *Flavivirus* and *Alphavirus* respectively, share several common characteristics and suffer, with small differences, the same changes to promote the membrane fusion between the virus and the target cell. The E and E1 suffer a significant conformational change that promote the DIII to move close the fusion loop, the DIII domain maintain the link of the fusion and the transmembrane domains. After the conformational change and reorganization of E and E1 protein, the DII domain is exposed and allows it to interact with the target membrane and promote the fusion. 

### Flavivirus replication mechanism

**Figure 2:**
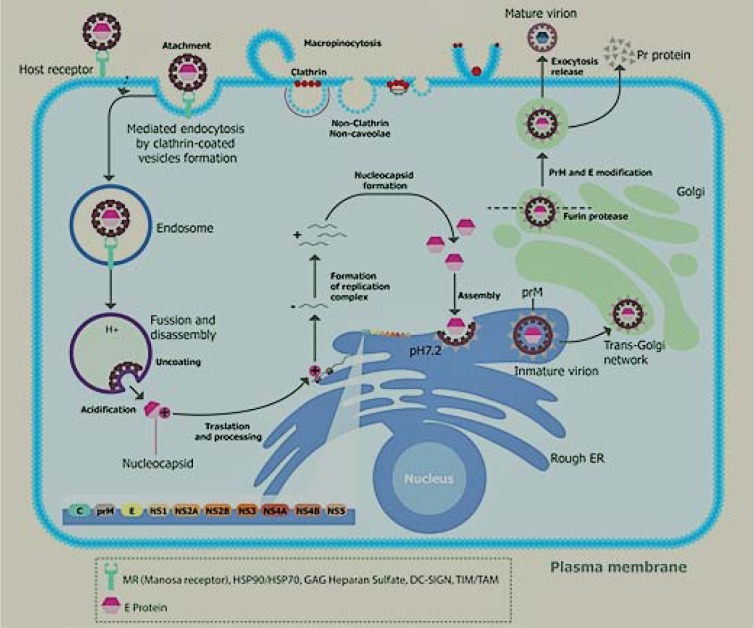
Life cycle of DENV in infected cells. The viral particles are released in the bloodstream; they then infect the white cells; the specific binding site of the virus is located in the E protein; it interacts with the specific superficial receptors of the host's cell, for example, mannose receptor, HSP90, HSP70, GAG heparin sulfate, DC-Sign, and TIM/TAM (Perera-Lecoin et al. 2013); this initiates the entry of the virus, endocytosis mediated by receptors is the primary route by which flavivirus are internalized. A summary of the steps that occur during the infection process from the entry of virus into the cell up to the release of new mature viral particles is as follows: The entry into the cell is achieved by endocytosis within clathrin-coated vesicles.The acidification of endosomes leads to insertion of the E protein into the endosomal membrane, the fusion of the viral envelope, and endosomal membrane releases nucleocapsid into the cytosol. After the nucleocapsid is uncovered, the viral RNA is translated as a single polyprotein, translocated across the host endoplasmic reticulum membrane.Particles that have budded from the ER are then processed by carbohydrate addition and modification, as they proceed through the Golgi membrane system. It is likely that transportation into the *trans*-Golgi network requires the presence of the glycosylated prM protein. The mature viral particles are released by exocytosis and can infect more cells.

Once the flaviviruses are in the endosomes; the acidic pH triggers the assembly of E trimers that leads to the release of the nucleocapside into the cell cytoplasm. Subsequently the capsid-RNA complex leaves the endosome, presumably through the pores (Figure 2(Fig. 2)). The RNA produces a polyprotein, which is directed to the endoplasmic reticulum, where it is processed by cellular and virus-derived proteases into structural and non-structural proteins (Rodenhuis-Zybert et al., 2010[68]; Garcia-Blanco et al., 2016[26]).

The E and prM proteins are glycosylated and then the NS proteins trigger viral genome replication. Several modifications of viral RNA are observed during its replication, such as methylation, which protects the RNA from quick degradation and manages the transcription efficiency. This modification is necessary for the formation of the virions and it is detected in different flaviviruses (Yap et al., 2017[101]). 

The coupling of protein synthesis, RNA synthesis, and the virion assembly on membranous structures assures that the newly synthesized RNA genome can be associated with C protein, thus initiating the assembly process. RNA encapsidation initiates the budding of particles into the ER-derived membrane vesicles, formation of which is believed to be induced by prM/E heterodimers (Welsch et al., 2009[97]; Rodenhuis-Zybert et al., 2010[68]). These immature virions that have budded into the ER, are then processed by carbohydrate addition and modification as they proceed through the Golgi membrane system. It is likely that the transportation through the* trans*-Golgi network requires the presence of the glycosylated prM protein, which is obtained after prM/E heterodimers dissociation. prM is cleaved, just prior virus release, by the host-encoded furin, into a pr peptide and a membrane-associated M protein, allowing for the cleavage of E proteins and thus helping in the virion maturation. Virions follow the exocytosis pathway and are released into the extracellular space by fusion of vesicles containing virions with the plasma membrane (Keelapang et al., 2004[33]; Rodenhuis-Zybert et al., 2010[68]). Mature viruses are released from the host cell by exocytosis and the new mature virus can infect more cells (Suwanmanee and Luplertlop, 2017[81]). 

### Alphavirus replication mechanism

**Figure 3:**
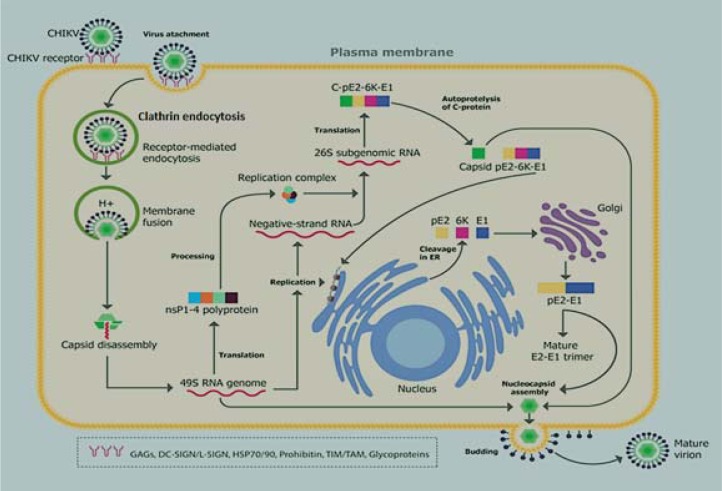
Life cycle of CHIKV in infected cells. The majority of the cells that get infected by CHIKV express glycosaminoglycans that apparently act as a virus-cell binding factor, increasing infectivity. Once endocytosed inside the endosome, there is a reduction in pH level. Then the endosome releases the nucleocapsid and the genome; the nsP123 precursor is translated from the viral genome and it binds to free nsP4 along with some host proteins to form the replication complex. When nsP123 concentration is enough to support an efficient reaction, it is cleaved into mature non-structural proteins: nsP1-4. These proteins together with the host cell proteins act as plus-strand RNA replicase. The 26S sub-genomic RNA encodes for the polyprotein precursor for structural proteins, which is cleaved by an autoproteolytic serine protease in order to yield C, pE2, 6K, and E1. The C and 6K proteins are accumulated in the cell's cytoplasm for the formation of new nucleocapsids. The E3, E2, and E1 proteins are glycosylated at endoplasmic reticulum and then are sent, through the Golgi apparatus, in vesicles to the cell membrane, where can interact with the nucleocapsid, resulting in the viral particles maturation to be finally released by exocytosis.

After internalization of alphaviruses in endosomes, the reduction in pH level, due to the H^+^ pump, leads to the conformational reorganization of viral heterodimer E1-E2, releasing E1 as a result. Thereby, the domain II is exposed and the viral particle fuses with the membrane of the host cell (Schuffenecker et al., 2006[70]). The E3 protein interacts with the E2 protein, stabilizing a region called the “acid-sensitive region”, indirectly facilitating the activation of E1 that is required for the membrane fusion (Sjöberg et al., 2011[78]). The endosome releases the nucleocapsid and the RNA genome; both coupled by interaction of the major RNA subunit 60S with the signal protein of the nucleocapsid. This mechanism is highly conserved among alphaviruses (Sjöberg et al., 2011[78]) (Figure 3(Fig. 3)).

Subsequently the genome is translated to generate the non-structural and structural proteins. The non-structural proteins (nsP1-4) and their precursors are necessary for the replication of the viral RNA. The five structural proteins (C, E3, E2, 6K, E1) and their precursors are necessary for viral encapsidation and budding. Viral replication occurs in the host cytoplasm in close connection with the Golgi apparatus (Thiberville et al., 2013[85]).

The non-structural proteins (nsP1-4) are translated as polyprotein: P123 and P1234 (in smaller proportion) (Solignat et al., 2009[79]). First nsP4 is cleaved off from nsP123 to form the replication complex, which produces the negative strand (Barton et al., 1991[8]); this is detected during the early phases of CHIKV replication. When nsP123 concentration is enough to support an efficient reaction, it is cleaved into mature nsP1, nsP2, nsP3, and nsP4 (Solignat et al., 2009[79]). These proteins, together with host cell proteins, act as a plus-strand RNA replicase, which produces the 26S sub-genomic plus-strand RNA using the negative-strand RNA as a template (Shirako and Strauss, 1994[74]). This 26S sub-genomic RNA encodes the polyprotein precursor for structural proteins, which is cleaved to yield C, pE2, 6K, and E1 by an autoproteolytic serine protease (Solignat et al., 2009[79]). C and 6K proteins are accumulated in cell's cytoplasm for the formation of new nucleocapsids, which are assembled with a copy of the viral genome by means of proteolytic cuts. E3, E2, and E1 proteins, are post-transcriptionally modified and initiate their glycosylated in the endoplasmic reticulum and translocated to the Golgi apparatus and pack in vesicles to be delivered in the cell membrane. When the nucleocapsid interacts with the glycoproteins accumulated in the cell's membrane, the viral particles under the maturation process acquire a membrane envelope and then they are finally released by exocytosis (Bautista-Reyes et al., 2017[9]).

### Flavivirus and alphavirus treatments

To date there is not a specific treatment to eradicate the infection by arboviruses, the treatment choice is basically to reduce the symptoms, although, the viral replication is not affected. A vaccine for DENV has been recently implemented to be used in people aged 9 to 45 years living in endemic areas (Barrows et al., 2016[7]). However, there are limitations to the application of this vaccine, since it has been reported that people who have never had dengue are more susceptible to developing a more severe disease, if they are vaccinated. In addition, the Philippine government suspended the use of the vaccine after the death of three people due to dengue despite being vaccinated (Dyer, 2017[22]). Therefore, there is no vaccine available for the treatment of DENV (Lim et al., 2013[42]).

In the middle of this bleak panorama, several candidates for arbovirus vaccines have been proposed, however, these are only tested in animal models. For example, Weger-Lucarelli and colleagues (2014[96]) describe the construction and characterization of a modified Ankara virus-based vaccine, and expressing CHIKV E3 and E2 proteins, the authors demonstrated that the vaccine protected different type of mice against CHIKV infection (Weger-Lucarelli et al., 2014[96]). Prow and colleagues, described a single-vector construct-based vaccine, that expresses the prME protein of zika virus and the polyprotein conformed by C-E3-E2-6K-E1 proteins of chikungunya virus, having as a result that a single dose of the vaccine in different type of mice, can elicit neutralizing antibodies, therefore, it provides protection against both viruses (Prow et al., 2018[63]). 

The use of natural products, as therapeutic tools, is an ancestral practice in different cultures. According to WHO 80 % of the population of some countries from Africa and Asia depend on the use of traditional medicines to deal with some diseases (Oliveira et al., 2017[56]). The neglected disease, as some of the mentioned in this work, required the urgent description of new molecules with antiviral capacity for their treatment, as a sample; there are multiple articles that describe the use, *in vitro,* of natural products as inhibitors of some arboviruses infection. 

For instance, a compilation of studies, from 1997 to 2012, describes the use of natural products of 31 species (that belong to 24 different plant families). Those studies include the analysis of species as Cladosiphon okamuranus, Leucaena leucocephala, Mimosa scabrella, Tephrosia madrensis, Cryptonemia crenulata, Gymnogongrus griffithsiae, Meristiella gelidium, among others. From which, were isolated approximately ten phytochemicals: as Fucoidan, Galactomannan, Glabranine, 7-O-methyl-glabranina, Galactane, hyperóeside, Kappa carragenane, Zosterice acid, 4-hydroxypanduratine A, panduratine A. These compounds belong to various kinds of chemical families such as: polysaccharides sulfated, flavonoids, quercetine and natural compounds of chalcone, which have anti-proliferative activity against dengue virus (Abd Kadir et al., 2013[1]). 

In relation to zika, curcumin, a common food additive, was considered capable of reducing ZIKV infectivity by preventing the virus binding to cells, without showing adverse effects on cell viability (Sharma et al., 2018[71]). Quercetine, a flavonoid present in fruits, vegetables, leaves and grains, inhibits the enzymatic activity of NS2B-NS3 in a dose-dependent manner. Also, it has been reported that commercially available quercetine inhibits ZIKV protease with an IC_50_ of 26.0 ± 0.1 μM (Mounce et al., 2017[49]). Although all these studies are promising, more researches are still needed, in order to validate them as therapeutic tools. 

Despite the fact the use of natural products to inhibit different viruses has proven to be feasible and with good results *in vitro*. The knowledge of the biology of *Flavivirus* and *Alphavirus *have accelerated the discovery of selective targets to attack, with possibilities to inhibit their replication. In the case of ZIKV these proteins are well known as possible targets: NS2B-NS3 protease, NS3 helicase, NS5 polymerase, NS5 methyltransferase, the envelope and the capsid. To DENV, the research has been focused on multifunctional enzymes NS3, NS5 and the capsid protein. In the case of CHIKV one of the most suitable targets is the cysteine protease domain nsP2. All these proteins were selected as target because of their important role in the respective viral cycle replication.

However, Roy et al. (2017[69]) assessed a set of 9 natural products against zika virus, their results show that 6 of these compounds may inhibit the virus replication and have interaction as no competitive inhibitors of the NS2B-NS3 protein. In another study, that involved over 1350 extracts of plants as inhibitors of the DENV polymerase using the domain RdRp of DENV-2 NS5, 49 extracts showed an inhibition of at least 80 % infection, and one of these extracts, obtained from *Cryptocarya chartacea* exhibited 90 % inhibition at a concentration of 10 µg/ml. Subsequent purification of this extract reported 10 compounds: 1 pinocembrin and 9 chartaceone derivatives of pinocembrin. The pinocembrin did not show antienzymatic activity, however all the chartaceone derivatives showed different values of inhibition (Allard et al., 2011[4]). 

Pohjala et al. evaluated 356 (123 natural products and 233 clinical approved drugs) as inhibitors of CHIKV infection, using a stable replication cell line. Their results showed that under experimental conditions the flavonoids apigenin, chrysin, naringenin and silybin, were the most actives against CHIKV (Pohjala et al., 2011[60]). The Harringtonine, a natural product obtained from *Cephalotaxus sp*, shows a high antiproliferative activity against CHIKV, with EC_50 _of 0,24 µM and a very low cytotoxicity. These results suggest a possible inhibition at early stages of viral replication; additionally this compound was able to inhibit the replication of Sindbis virus too, suggesting a possible inhibition of other alphaviruses (Kaur et al., 2013[32]). In accordance with the previously indicated and, also, including other authors who have made relevant contributions on this subject, Table 1(Tab. 1) (References in Table 1: Brecher et al., 2017[14]; Carneiro et al., 2016[16]; Kaur et al., 2013[32]; Khan et al., 2010[35]; Khan et al., 2011[34]; Kiat et al., 2006[36]; Oliveira et al., 2017[56]; Rausch et al., 2017[67]; Roy et al., 2017[69]) shows the main natural products that have been evaluated against *Flavivirus* and *Alphavirus.*

## Concluding Remarks

Arbovirus, such as dengue, zika, and chikungunya viruses, are an important threat to global public health, especially in subtropical and tropical regions where these are maintained due to the peculiar environmental conditions which favor the development and proliferation of their main vectors, the *Aedes aegypti* and *Aedes albopictus. *The vector control strategies have failed, mainly due to insecticides resistance (Suwanmanee and Luplertlop, 2017[81]); in addition to this, there are factors such as the low coverage and efficiency of basic hygiene services in developing countries. This environment facilitates a favorable and permanent culture medium for the cyclical prevalence of these diseases that are still present in most Latin American countries (PAHO-CHA-IR, 2016[57]; Higuera and Ramírez, 2019[29]).

The discovery and/or design of new medicines to treat some of the diseases caused by arbovirus are a great challenge, but also an urgent need. To date there is no specific treatment for infection caused by DENV, ZIKV, CHIKV and other human-related infections caused by arbovirus. The development of a vaccine is still ongoing, but that directed against DENV has not shown safety results. Despite the enormous difficulties for the development of new vaccines that can be effective against communicable diseases, in the present case of arboviral origin, this strategy continues being the most important alternative to prevent and counteract the negative impact on global public health of the above pathologies.

On the other hand, the existence of some promising natural products and their possible therapeutic targets of action were stated. Highlighting that for DENV and ZIKV the NS2B.NS3 protease coincides as a potential target, further Tigocherin A and Tigocherriolide A showed potential inhibitory activity for DENV, having as a possible target of action the NS5RdRp protein. Nevertheless, in the case of CHIKV still is not clear a possible action target, however, it is known that the first stage of the replication cycle is the most susceptible to the extracts tested (Khan et al., 2011[34]).

Despite the extensive knowledge of the molecular structure and replication mechanisms of these arboviruses, there are still many gaps in what we know. Future investigations are necessary to elucidate many molecular aspects that can give us insights into the viral emergence mechanisms, cellular pathogenesis, and host-virus interactions. These would be helpful for the development of urgent control strategies and for improving their effectiveness. 

## Notes

Anny Karely Rodriguez and Felio Bello (Faculty of Agricultural and Livestock Sciences, Program of Veterinary Medicine, Universidad de La Salle, Bogotá, 110131, Colombia; Tel:+ 57-313-4213616, E-mail: felbello@unisalle.edu.co) contributed equally as corresponding authors.

## Acknowledgements

The authors would like to thank Vice-rectory of Science Technology and Research (VCTI) of the Universidad Antonio Nariño (research grant # 2017230) and Colciencias (research grant cod. 124380664546 and research grant cod. 325672553402). 

## Conflict of interest

The authors have no conflict of interest to report.

## Figures and Tables

**Table 1:**
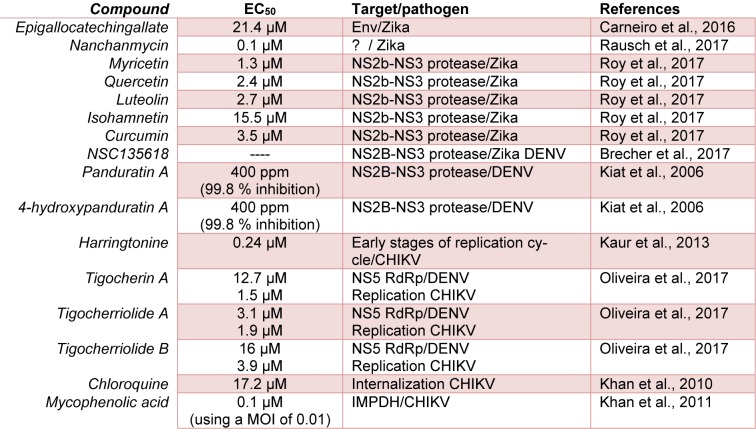
Summary about some naturals products which has been evaluated against Flaviviruses and Alphaviruses

## References

[1] Abd Kadir SL, Yaakob H, Mohamed Zulkifli R (2013). Potential anti-dengue medicinal plants: a review. J Nat Med.

[2] Abu Bakar F, Ng LFP (2018). Nonstructural proteins of alphavirus-potential targets for drug development. Viruses.

[3] Alayli F, Scholle F (2016). Dengue virus NS1 enhances viral replication and pro-inflammatory cytokine production in human dendritic cells. Virology.

[4] Allard P-M, Dau ETH, Eydoux C, Guillemot J-C, Dumontet V, Poullain C (2011). Alkylated flavanones from the bark of Cryptocarya chartacea as dengue virus NS5 polymerase inhibitors. J Nat Prod.

[5] Allison SL, Schalich J, Stiasny K, Mandl CW, Kunz C, Heinz FX (1995). Oligomeric rearrangement of tick-borne encephalitis virus envelope proteins induced by an acidic pH. J Virol.

[6] Baronti C, Sire J, de Lamballerie X, Quérat G (2010). Nonstructural NS1 proteins of several mosquito-borne Flavivirus do not inhibit TLR3 signaling. Virology.

[7] Barrows NJ, Campos RK, Powell ST, Prasanth KR, Schott-Lerner G, Soto-Acosta R (2016). A screen of FDA-approved drugs for inhibitors of zika virus infection. Cell Host Microbe.

[8] Barton DJ, Sawicki SG, Sawicki DL (1991). Solubilization and immunoprecipitation of alphavirus replication complexes. J Virol.

[9] Bautista-Reyes E, Núñez-Avellaneda D, Alonso-Palomares LA, Salazar MAI (2017). Chikungunya: Molecular aspects, clinical outcomes and pathogenesis. Rev Investig Clin.

[10] Bhatt S, Gething PW, Brady OJ, Messina JP, Farlow AW, Moyes CL (2013). The global distribution and burden of dengue. Nature.

[11] Blitvich BJ, Firth AE (2017). A review of flaviviruses that have no known arthropod vector. Viruses.

[12] Bollati M, Alvarez K, Assenberg R, Baronti C, Canard B, Cook S (2010). Structure and functionality in flavivirus NS-proteins: Perspectives for drug design. Antiviral Res.

[13] Brand C, Bisaillon M, Geiss BJ (2017). Organization of the flavivirus RNA replicase complex. Wiley Interdiscip Rev RNA.

[14] Brecher M, Li Z, Liu B, Zhang J, Koetzner CA, Alifarag A (2017). A conformational switch high-throughput screening assay and allosteric inhibition of the flavivirus NS2B-NS3 protease. PLoS Pathog.

[15] Brinton MA, Basu M (2015). Functions of the 3′ and 5′ genome RNA regions of members of the genus Flavivirus. Virus Res.

[16] Carneiro BM, Batista MN, Braga ACS, Nogueira ML, Rahal P (2016). The green tea molecule EGCG inhibits Zika virus entry. Virology.

[17] Cecchetto J, Fernandes FCB, Lopes R, Bueno PR (2017). The capacitive sensing of NS1 Flavivirus biomarker. Biosens Bioelectron.

[18] Chen H-R, Lai Y-C, Yeh T-M (2018). Dengue virus non-structural protein 1: a pathogenic factor, therapeutic target, and vaccine candidate. J Biomed Sci.

[19] Chen S, Wu Z, Wang M, Cheng A (2017). Innate immune evasion mediated by flaviviridae non-structural proteins. Viruses.

[20] Colón-González FJ, Peres CA, Steiner São Bernardo C, Hunter PR, Lake IR (2017). After the epidemic: Zika virus projections for Latin America and the Caribbean. Ekpo UF, editor. PLoS Negl Trop Dis.

[21] Douam F, Ploss A (2018). Yellow fever virus: knowledge gaps impeding the fight against an old foe. Trends Microbiol.

[22] Dyer O (2017). Philippines halts dengue immunisation campaign owing to safety risk. BMJ.

[23] El Sahili A, Lescar J (2017). Dengue virus non-structural protein 5. Viruses.

[24] Figueiredo LTM (2019). Human urban arboviruses can infect wild animals and jump to sylvatic maintenance cycles in South America. Front Cell Infect Microbiol.

[25] Ganesan V, Duan B, Reid S, Ganesan VK, Duan B, Reid SP (2017). Chikungunya virus: pathophysiology, mechanism, and modeling. Viruses.

[26] Garcia-Blanco MA, Vasudevan SG, Bradrick SS, Nicchitta C (2016). Flavivirus RNA transactions from viral entry to genome replication. Antiviral Res.

[27] Götte B, Liu L, McInerney G, Götte B, Liu L, McInerney GM (2018). The enigmatic alphavirus non-structural protein 3 (nsP3) revealing its secrets at last. Viruses.

[28] Hennessey M, Fischer M, Staples JE (2016). Zika virus spreads to new areas - region of the Americas, May 2015-January 2016. Am J Transplant.

[29] Higuera A, Ramírez JD (2019). Molecular epidemiology of dengue, yellow fever, Zika and Chikungunya arboviruses: An update. Acta Trop.

[30] Hubálek Z, Rudolf I, Nowotny N (2014). Arboviruses pathogenic for domestic and wild animals. Adv Virus Res.

[31] Jose J, Snyder JE, Kuhn RJ (2009). A structural and functional perspective of alphavirus replication and assembly. Future Microbiol.

[32] Kaur P, Thiruchelvan M, Lee RCH, Chen H, Chen KC, Ng ML (2013). Inhibition of chikungunya virus replication by harringtonine, a novel antiviral that suppresses viral protein expression. Antimicrob Agents Chemother.

[33] Keelapang P, Sriburi R, Supasa S, Panyadee N, Songjaeng A, Jairungsri A (2004). Alterations of pr-M cleavage and virus export in pr-M junction chimeric dengue viruses. J Virol.

[34] Khan M, Dhanwani R, Patro IK, Rao PVL, Parida MM (2011). Cellular IMPDH enzyme activity is a potential target for the inhibition of Chikungunya virus replication and virus induced apoptosis in cultured mammalian cells. Antiviral Res.

[35] Khan M, Santhosh SR, Tiwari M, Lakshmana Rao PV, Parida M (2010). Assessment of in vitro prophylactic and therapeutic efficacy of chloroquine against Chikungunya virus in vero cells. J Med Virol.

[36] Kiat TS, Pippen R, Yusof R, Ibrahim H, Khalid N, Rahman NA (2006). Inhibitory activity of cyclohexenyl chalcone derivatives and flavonoids of fingerroot, Boesenbergia rotunda (L.), towards dengue-2 virus NS3 protease. Bioorg Med Chem Lett.

[37] Kostyuchenko VA, Zhang Q, Tan JL, Ng T-S, Lok S-M (2013). Immature and mature dengue serotype 1 virus structures provide insight into the maturation process. J Virol.

[38] Leta S, Beyene TJ, De Clercq EM, Amenu K, Kraemer MUG, Revie CW (2018). Global risk mapping for major diseases transmitted by Aedes aegypti and Aedes albopictus. Int J Infect Dis.

[39] Leung JY, Pijlman GP, Kondratieva N, Hyde J, Mackenzie JM, Khromykh AA (2008). Role of nonstructural protein NS2A in flavivirus assembly. J Virol.

[40] Li L, Jose J, Xiang Y, Kuhn RJ, Rossmann MG (2010). Structural changes of envelope proteins during alphavirus fusion. Nature.

[41] Liang Q, Luo Z, Zeng J, Chen W, Foo S-S, Lee S-A (2016). Zika Virus NS4A and NS4B proteins deregulate Akt-mTOR signaling in human fetal neural stem cells to inhibit neurogenesis and induce autophagy. Cell Stem Cell.

[42] Lim SP, Wang Q-Y, Noble CG, Chen Y-L, Dong H, Zou B (2013). Ten years of dengue drug discovery: Progress and prospects. Antiviral Res.

[43] Lindenbach BD, Rice CM (2003). Molecular biology of flaviviruses. Adv Virus Res.

[44] Litvoc MN, Novaes CTG, Lopes MIBF, Litvoc MN, Novaes CTG, Lopes MIBF (2018). Yellow fever. Rev Assoc Med Bras.

[45] Liu Y, Liu J, Du S, Shan C, Nie K, Zhang R (2017). Evolutionary enhancement of Zika virus infectivity in Aedes aegypti mosquitoes. Nature.

[46] Malisheni M, Khaiboullina SF, Rizvanov AA, Takah N, Murewanhema G, Bates M (2017). Clinical efficacy, safety, and immunogenicity of a live attenuated tetravalent dengue vaccine (CYD-TDV) in children: A systematic review with meta-analysis. Front Immunol.

[47] Martina BEE, Koraka P, Osterhaus ADME (2009). Dengue virus pathogenesis: an integrated view. Clin Microbiol Rev.

[48] McLean JE, Wudzinska A, Datan E, Quaglino D, Zakeri Z (2011). Flavivirus NS4A-induced autophagy protects cells against death and enhances virus replication. J Biol Chem.

[49] Mounce BC, Cesaro T, Carrau L, Vallet T, Vignuzzi M (2017). Curcumin inhibits Zika and chikungunya virus infection by inhibiting cell binding. Antiviral Res.

[50] Murray CL, Jones CT, Rice CM (2008). Architects of assembly: roles of Flaviviridae non-structural proteins in virion morphogenesis. Nat Rev Microbiol.

[51] Musso D, Gubler DJ (2016). Zika virus. Clin Microbiol Rev.

[52] Mustafa MS, Rasotgi V, Jain S, Gupta V (2015). Discovery of fifth serotype of dengue virus (DENV-5): A new public health dilemma in dengue control. Med Journal, Armed Forces India.

[53] Mutso M, Morro A, Smedberg C, Kasvandik S, Aquilimeba M, Teppor M (2018). Mutation of CD2AP and SH3KBP1 binding motif in alphavirus nsP3 hypervariable domain results in attenuated virus. Viruses.

[54] Natarajan S (2010). NS3 protease from flavivirus as a target for designing antiviral inhibitors against dengue virus. Genet Mol Biol.

[55] Nemésio H, Palomares-Jerez F, Villalaín J (2012). NS4A and NS4B proteins from dengue virus: Membranotropic regions. Biochim Biophys Acta.

[56] Oliveira AF, Teixeira RR, Oliveira AS, Souza AP, Silva ML, Paula SO (2017). Potential antivirals: Natural products targeting replication enzymes of dengue and chikungunya viruses. Molecules.

[57] PAHO-CHA-IR (2016). Vector born diseases (VBD) in the region of the Americas. http://ais.paho.org/phip/viz/cha_cd_vectorborndiseases.asp.

[58] Perera-Lecoin M, Meertens L, Carnec X, Amara A (2013). Flavivirus entry receptors: an update. Viruses.

[59] Pietilä MK, Hellström K, Ahola T (2017). Alphavirus polymerase and RNA replication. Virus Res.

[60] Pohjala L, Utt A, Varjak M, Lulla A, Merits A, Ahola T (2011). Inhibitors of alphavirus entry and replication identified with a stable Chikungunya replicon cell line and virus-based assays. PLoS One.

[61] Potisopon S, Priet S, Collet A, Decroly E, Canard B, Selisko B (2014). The methyltransferase domain of dengue virus protein NS5 ensures efficient RNA synthesis initiation and elongation by the polymerase domain. Nucleic Acids Res.

[62] Prompetchara E, Ketloy C, Thomas SJ, Ruxrungtham K (2019). Dengue vaccine: Global development update. Asian Pacific J Allergy Immunol.

[63] Prow NA, Liu L, Nakayama E, Cooper TH, Yan K, Eldi P (2018). A vaccinia-based single vector construct multi-pathogen vaccine protects against both Zika and chikungunya viruses. Nat Commun.

[64] Puerta-Guardo H, Glasner DR, Harris E (2016). Dengue virus NS1 disrupts the endothelial glycocalyx, leading to hyperpermeability. PLoS Pathog.

[65] Rana J, Gulati S, Rajasekharan S, Gupta A, Chaudhary V, Gupta S (2017). Identification of potential molecular associations between chikungunya virus non-structural protein 2 and human host proteins. Acta Virol.

[66] Rastogi M, Sharma N, Singh SK (2016). Flavivirus NS1: a multifaceted enigmatic viral protein. Virol J.

[67] Rausch K, Hackett BA, Weinbren NL, Reeder SM, Sadovsky Y, Hunter CA (2017). Screening bioactives reveals nanchangmycin as a broad spectrum antiviral active against Zika virus. Cell Rep.

[68] Rodenhuis-Zybert IA, Wilschut J, Smit JM (2010). Dengue virus life cycle: viral and host factors modulating infectivity. Cell Mol Life Sci.

[69] Roy A, Lim L, Srivastava S, Lu Y, Song J (2017). Solution conformations of Zika NS2B-NS3pro and its inhibition by natural products from edible plants. PLoS One.

[70] Schuffenecker I, Iteman I, Michault A, Murri S, Frangeul L, Vaney M-C (2006). Genome microevolution of Chikungunya viruses causing the Indian ocean outbreak. PLoS Med.

[71] Sharma R, Kesari P, Kumar P, Tomar S (2018). Structure-function insights into chikungunya virus capsid protein: Small molecules targeting capsid hydrophobic pocket. Virology.

[72] Shi Y, Gao GF (2017). Structural biology of the Zika virus. Trends Biochem Sci.

[73] Shin G, Yost SA, Miller MT, Elrod EJ, Grakoui A, Marcotrigiano J (2012). Structural and functional insights into alphavirus polyprotein processing and pathogenesis. Proc Natl Acad Sci U S A.

[74] Shirako Y, Strauss JH (1994). Regulation of Sindbis virus RNA replication: uncleaved P123 and nsP4 function in minus-strand RNA synthesis, whereas cleaved products from P123 are required for efficient plus-strand RNA synthesis. J Virol.

[75] Silva LA, Dermody TS (2017). Chikungunya virus: epidemiology, replication, disease mechanisms, and prospective intervention strategies. J Clin Invest.

[76] Silva LA, Khomandiak S, Ashbrook AW, Weller R, Heise MT, Morrison TE (2014). A single-amino-acid polymorphism in Chikungunya virus E2 glycoprotein influences glycosaminoglycan utilization. J Virol.

[77] Simon F, Javelle E, Oliver M, Leparc-Goffart I, Marimoutou C (2011). Chikungunya virus infection. Curr Infect Dis Rep.

[78] Sjöberg M, Lindqvist B, Garoff H (2011). Activation of the alphavirus spike protein is suppressed by bound E3. J Virol.

[79] Solignat M, Gay B, Higgs S, Briant L, Devaux C (2009). Replication cycle of chikungunya: A re-emerging arbovirus. Virology.

[80] Song H, Qi J, Haywood J, Shi Y, Gao GF (2016). Zika virus NS1 structure reveals diversity of electrostatic surfaces among flaviviruses. Nat Struct Mol Biol.

[81] Suwanmanee S, Luplertlop N (2017). Dengue and Zika viruses: lessons learned from the similarities between these Aedes mosquito-vectored arboviruses. J Microbiol.

[82] Tambonis T, Contessoto VG, Bittar C, Calmon MF, Nogueira ML, Rahal P (2017). Asian lineage of Zika virus RNA pseudoknot may induce ribosomal frameshift and produce a new neuroinvasive protein ZIKV-NS1’. bioRxiv.

[83] Taylor A, Liu X, Zaid A, Goh LYH, Hobson-Peters J, Hall RA (2017). Mutation of the N-terminal region of Chikungunya virus capsid protein: Implications for vaccine design. MBio.

[84] Teo CSH, Chu JJH (2014). Cellular vimentin regulates construction of dengue virus replication complexes through interaction with NS4A protein. J Virol.

[85] Thiberville S-D, Moyen N, Dupuis-Maguiraga L, Nougairede A, Gould EA, Roques P (2013). Chikungunya fever: Epidemiology, clinical syndrome, pathogenesis and therapy. Antiviral Res.

[86] Tomar S, Hardy RW, Smith JL, Kuhn RJ (2006). Catalytic core of alphavirus nonstructural protein nsP4 possesses terminal adenylyltransferase activity. J Virol.

[87] Torres JR, Orduna TA, Piña-Pozas M, Vázquez-Vega D, Sarti E (2017). Epidemiological characteristics of Dengue disease in Latin America and in the Caribbean: A systematic review of the literature. J Trop Med.

[88] Uno N, Ross TM (2018). Dengue virus and the host innate immune response. Emerg Microbes Infect.

[89] Volk DE, Lee Y-C, Li X, Thiviyanathan V, Gromowski GD, Li L (2007). Solution structure of the envelope protein domain III of dengue-4 virus. Virology.

[90] Volk DE, May FJ, Gandham SHA, Anderson A, Von Lindern JJ, Beasley DWC (2009). Structure of yellow fever virus envelope protein domain III. Virology.

[91] Vu DM, Jungkind D, LaBeaud AD (2017). Chikungunya Virus. Clin Lab Med.

[92] Wang A, Thurmond S, Islas L, Hui K, Hai R (2017). Zika virus genome biology and molecular pathogenesis. Emerg Microbes Infect.

[93] Wang D, Chen C, Liu S, Zhou H, Yang K, Zhao Q (2017). A mutation identified in neonatal microcephaly destabilizes Zika virus NS1 assembly in vitro. Sci Rep.

[94] Wang H, Han M, Qi J, Hilgenfeld R, Luo T, Shi Y (2017). Crystal structure of the C-terminal fragment of NS1 protein from yellow fever virus. Sci China Life Sci.

[95] Weaver SC, Reisen WK (2010). Present and future arboviral threats. Antiviral Res.

[96] Weger-Lucarelli J, Chu H, Aliota MT, Partidos CD, Osorio JE (2014). A novel MVA vectored Chikungunya virus vaccine elicits protective immunity in mice. PLoS Negl Trop Dis.

[97] Welsch S, Miller S, Romero-Brey I, Merz A, Bleck CKE, Walther P (2009). Composition and three-dimensional architecture of the dengue virus replication and assembly sites. Cell Host Microbe.

[98] Xia H, Luo H, Shan C, Muruato AE, Nunes BTD, Medeiros DBA (2018). An evolutionary NS1 mutation enhances Zika virus evasion of host interferon induction. Nat Commun.

[99] Xie X, Zou J, Puttikhunt C, Yuan Z, Shi P-Y (2015). Two distinct sets of NS2A molecules are responsible for Dengue virus RNA synthesis and virion assembly. J Virol.

[100] Yap ML, Klose T, Urakami A, Hasan SS, Akahata W, Rossmann MG (2017). Structural studies of Chikungunya virus maturation. Proc Natl Acad Sci U S A.

[101] Yap S, Nguyen-Khuong T, Rudd PM, Alonso S (2017). Dengue virus glycosylation: What do we know?. Front Microbiol.

[102] Zhang X, Jia R, Shen H, Wang M, Yin Z, Cheng A (2017). Structures and functions of the envelope glycoprotein in flavivirus infections. Viruses.

[103] Zmurko J, Neyts J, Dallmeier K (2015). Flaviviral NS4b, chameleon and jack-in-the-box roles in viral replication and pathogenesis, and a molecular target for antiviral intervention. Rev Med Virol.

[104] Zou J, Xie X, Wang Q-Y, Dong H, Lee MY, Kang C (2015). Characterization of dengue virus NS4A and NS4B protein interaction. J Virol.

